# Measurement and analysis of the longitudinal level irregularity of the track beam in monorail tour-transit systems

**DOI:** 10.1038/s41598-022-23805-6

**Published:** 2022-11-10

**Authors:** Fengqi Guo, Pengjiao Wang

**Affiliations:** School of Civil Engineering, Central South University, Changsha, 410075 China

**Keywords:** Civil engineering, Mechanical engineering

## Abstract

Notable effects on the vertical dynamic response of vehicle-bridge systems are introduced by longitudinal level irregularity (LLI). However, in monorail systems, the measured data and associated spectrum of the LLI, i.e., the distribution curve of power spectral density versus wavelength, have not been reported in detail. To address this issue, we propose the inclination correction method (ICM) to measure the LLI in monorail tour-transit systems and further estimate and fit the spectral curves for the first time. The measuring principle of ICM is thoroughly described, and ICM is compared with conventional chord-based methods. In addition, the trend components of the measured LLI are eliminated through an adaptive method preceding spectral estimation to avoid potential errors. Notably, a simulation program is designed, and the results from existing methods are adopted to verify the proposed ICM. Based on an analysis of the results, the accuracy and robustness of ICM are demonstrated, and the applicability and advantages of autoregressive models and the proposed fractional functions in spectral analysis are revealed. Finally, combining qualitative and quantitative calculations, an evaluation approach for the generic spectra of monorail LLI is established.

## Introduction

**Figure 1:**
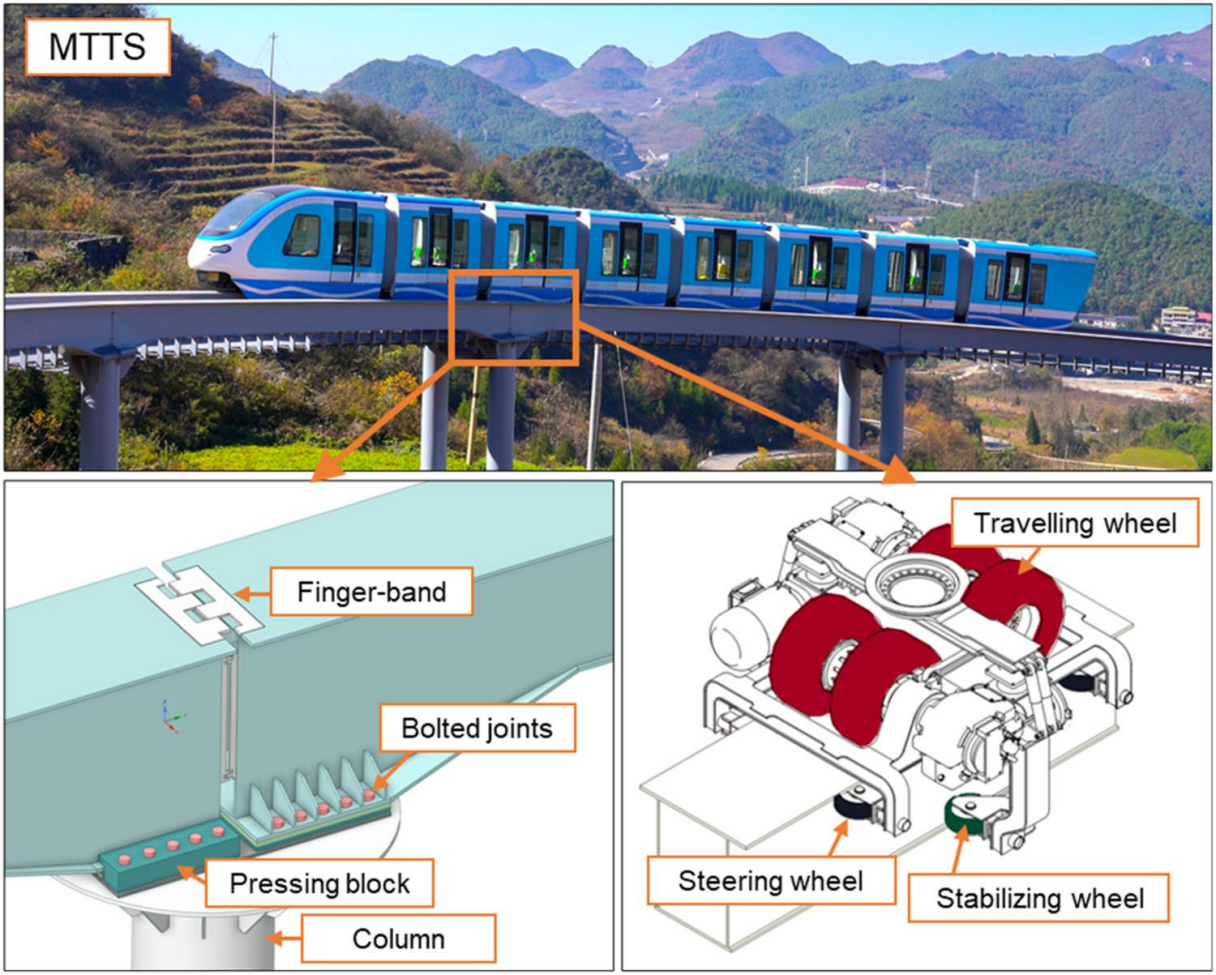
Configuration of the track beam and bogie of the monorail vehicle in MTTS.

The monorail tour-transit system (MTTS) is a new solution for scenic transportation. Over thirty MTTS projects have been developed or designed in China, while several projects are under construction or planning in South Korea, Malaysia, and Vietnam^1^. Similar to monorail systems in urban traffic^2^, MTTS utilizes a straddle-type monorail with travelling, steering, and stabilizing wheels that are in contact with the track beam during operation. Nevertheless, in MTTS, steel track beams are generally employed rather than (prestressed) concrete track beams. These beams are fastened by bolted joints on one side and restrained on the other by the pressing block, except for the direction along the track (see Fig. 1). On the one hand, this unique running mode provides high-level security, strong line adaptability, and environmental friendliness. On the other hand, a flexible track beam may bring challenges in controlling track irregularities (TIs) induced by various factors, such as the manufacturing process, cyclic loads, structural deformation or differential settlement^3^. In addition, structural construction in MTTS, which includes a high live load and complicated alignment settings, may simultaneously enhance TI-induced effects. Hence, attention should be given to TI issues in generic monorail systems.

It is well known in rail systems that the dynamic performance of vehicle and bridge (i.e., the track beam for the monorail) systems is poor if the track profile status is poor^4,5^. This work mainly focuses on the longitudinal level irregularity (LLI) of the track beam because of the fine stability of the straddle-type monorail in the lateral direction. The only available LLI data in monorail systems, which were used during extensive research on diverse issues, comes from the measurement of a 34.8 m long track beam in Japan in 2006^6^. In addition, road or railway profiles were adopted in several studies^7–9^ (as a substitute for or in comparison with the monorail spectrum), and the strong impact of LLI/TI on monorail systems was revealed. According to Ref.^8^, the calculation results of the vertical Sperling index indicated poor riding comfort when the amplitude of the irregularity reached a certain roughness level, and it was claimed that the surface roughness of the track beam should be strictly controlled for comfort purposes. Similar results were found in a suspension-type monorail system^9^, affirming that TI significantly influences the dynamic responses of both vehicle and bridge systems. Additionally, LLI has been considered in the simulation models of some studies, and dynamic analyses have been conducted in various fields. Gou et al.^10^, for instance, employed the Japanese spectrum and studied the dynamic response of bridge subsystems and the riding comfort in monorail subsystems. Wang et al.^11^ explored the effect of the connection stiffness on the vertical and lateral vibration amplitudes of a composite track beam. Based on the finite element method and multibody dynamics, Zhou et al.^12^ proposed a full-scale coupling model to evaluate the dynamic behaviour of a monorail vehicle. Notably, credible simulation results with LLI were obtained in these works when compared to field tests, although the exact influence of LLI on dynamic systems was not reported.

**Figure 2:**
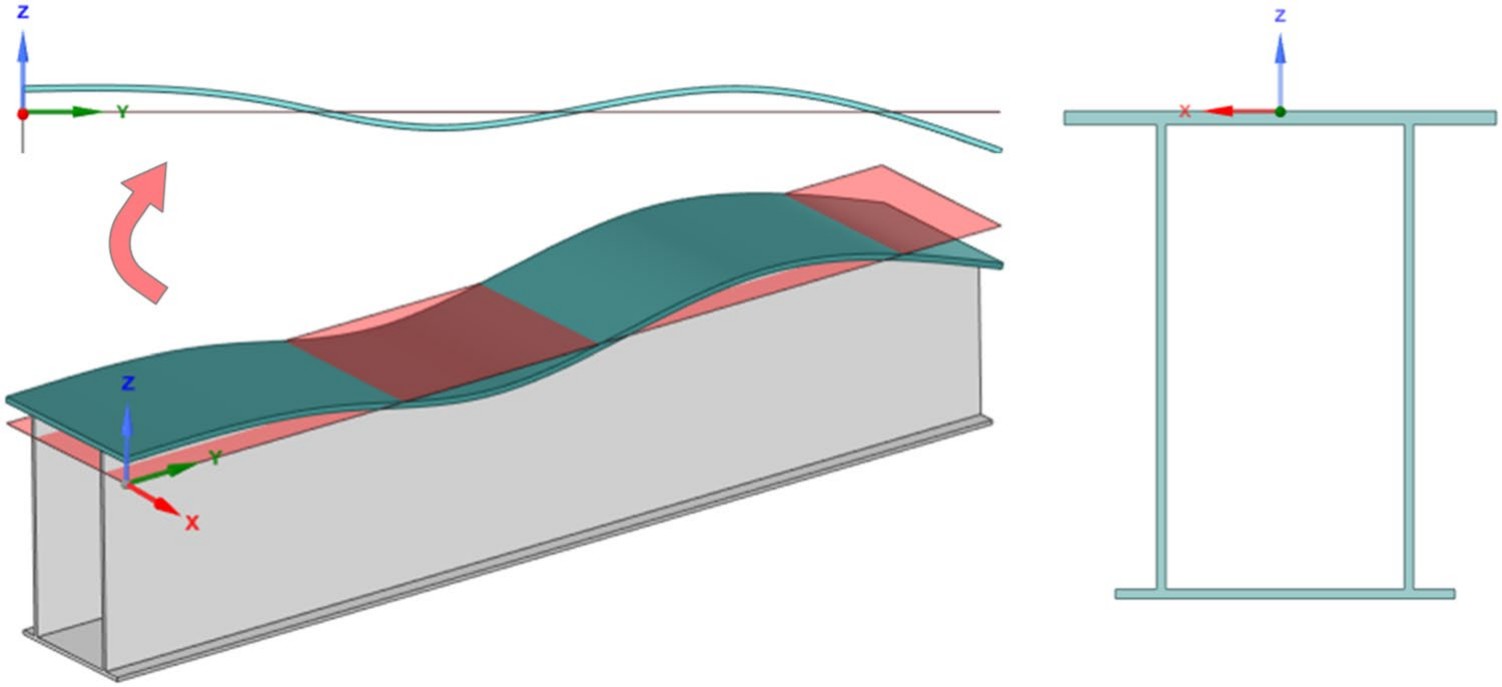
Definition of the LLI $(Z(y))$ and cross-section of the track beam.

As previously mentioned, contrary to the lack of research on the measurement of LLI, it is clear that the reliability of the LLI data has a significant impact on the accuracy of monorail system calculations. In principle, TIs are defined as the deviation of the track profile along different directions. On this basis, the distribution curve of LLI is defined as the deviation between the actual and designed elevations of the top plate along the track beam, as shown in Fig. 2. For efficient application, the power spectral density (PSD) versus wavelength curve (known as the LLI spectrum) is highly recommended to describe the characteristics of nonstationarity and randomness^13^. Different regions (e.g., the US, Germany, and China) have developed their own standard spectra of track irregularity for railway systems based on detailed measurements. For a monorail system, however, the sample size of available data is too small to reflect the randomness and nonstationarity of the LLI signal, and hence, the accuracy of the proposed spectrum^6^ may be affected. In fact, the length of track for LLI spectrum calculations should be longer than five times its maximum wavelength (generally 30 to 100 m in related studies^14,15^) to ensure the accuracy of the PSD and reduce the deviation error^16^. In summary, reviews of current research clearly indicate the urgent requirement of field-measured data of monorail track irregularity, but the available information is still sparse.

The lack of comprehensive instruments is one principal reason for the lack of field-measured research. In the literature, significant research^17–20^ has been reported on the design or application of measuring systems for railways; research on monorail systems, however, does not yet exist, and to the best of our knowledge, none of the above methods have been applied or verified for any monorail system. Generally, two main principles are adopted to measure the TIs, including LLI, i.e., the inertial reference method (IRM)^21^ and the chord measuring method (CMM, also known as the chord reference method)^22^. As highlighted in Ref.^23^, IRM systems are more easily affected by vibration factors such as the testing speed; thus, it may not be feasible for use with the relatively low-speed MTTS. In addition, the realization of IRM systems is more complicated and costly than that of CMM systems since the former usually integrate inertial sensors or equipment. Nevertheless, the CMM also has certain limitations, among which the fluctuation of the transfer function affects the irregular waveform and limited wavelength range of the measured signal. In addition, this effect cannot be permanently eliminated because of the amplitude oscillation of the transfer function; this part will be discussed later. Generally, traditional methods are not sufficient in this case.

To address the abovementioned deficiencies of the research on LLI for monorail systems, including the measurement method, field-measured data, and appropriate spectrum, we intend to take measurements of the LLI and perform analyses for practical MTTS projects. The main research is summarized as follows. (a) Regarding the lack of professional instruments and proper methods for monorail detection, we propose the inclination correction method (ICM), in which the LLI of the track beam is accurately measured using only basic instruments. (b) The measuring principle and its relationship with conventional methods are described in detail. Through a theoretical analysis and computer simulation, the accuracy of ICM is verified. In addition, the error synthesis of the ICM is explored, and an adaptive method is summarized to eliminate the trend components of the measured data preceding spectral analysis, which minimizes the measurement and calculation errors. (c) Furthermore, the PSD of the measured LLI is estimated and fitted by various approaches to obtain the monorail spectrum. Finally, a classification system of the monorail spectrum is preliminarily established by combining qualitative and quantitative calculations to consider track conditions at different levels and provide a favourable framework for future studies.

### Contributions and novelty

The main contributions and novelty of this work are summarized as follows. (a) We proposed the ICM, described its measuring principle and revealed its relationship with CMM for the first time. A theoretical analysis and calculation results from both simulation and field measurements validated the accuracy, applicability, and robustness of ICM in the detection of monorail systems and its advantages in terms of signal authenticity and wavelength range. (b) We conducted detailed measurements and analyses of the LLI of MTTS for the first time. A general fitting function was proposed to obtain the spectrum in a generic form, and a preliminary classification system was established. Compared to the only available spectrum, the sample size in this work is much larger, while the spectral estimation and fitting processes are more scientific. On this basis, the methods and case results presented in this work have extensive application value. (c) We introduced an adaptive method of trend extraction, which is an excellent supplement for ICM. In addition, it can be applied by itself in various fields of signal processing.

## Methodologies

### Measuring method for the LLI

**Figure 3:**
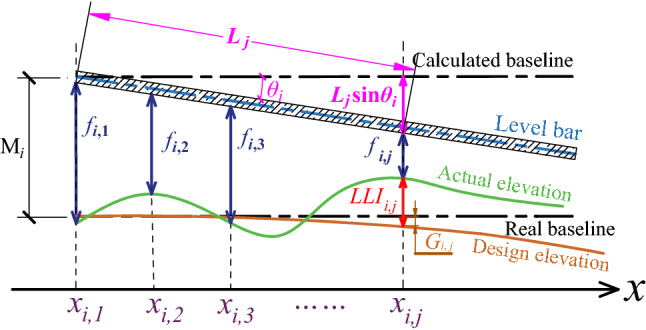
Measuring principle of the ICM (the $i{\text{-}}th$ segment).

As discussed previously, the IRM and CMM are two basic approaches for measuring the LLI in railway systems. However, the IRM is not applicable for monorail systems due to their low speed (a maximum speed of 10–40 km/h in different projects), while the CMM has deficiencies with respect to the variable transfer function. In addition, there is no professional equipment for track detection of monorail track beams. Therefore, we propose the ICM to measure the LLI. The measuring procedure of the ICM is conducted in successive segments, and the principle behind each measured segment is shown in Fig. 3 and described as follows.

Assume the green and orange lines are the actual and designed elevations of the track beam, respectively; then, the LLI, as we have defined, is the distance ${LLI}_{i,j}$ between them. The ‘real baseline’ is a ray extending from the starting point ${x}_{i,1}$, and the value of ${G}_{i,j}$ can be obtained from the design blueprint.

- Then, the ‘level bar’ is naturally placed on the top plate of the track beam, and the inclination of the level bar (${\theta }_{i}$) is measured by the high-precision inclinometer with a precision of 0.05° ± 1% and a resolution of 0.01°.
- Finally, the gap between the level bar and the track beam at each measuring point ${f}_{i,j}$ is measured by a feeler gauge (with a resolution of 0.01 mm). In addition, the measurement of the next segment is conducted exactly at the ending point of the previous segment.

The overall distribution curve of the LLI data can be obtained by the following process. For each segment, the distance between the calculated and real baseline (${M}_{i}$) is constant. Hence, we have Eq. (1), which relates the LLI of each measuring point to the first measuring point of the same segment. Furthermore, since the value of LLI is constant regardless of which segments it belongs to, a recursive relationship can be formed: $\langle {LLI}_{i,j}={LLI}_{i,1}-{C}_{i,j}\rangle$ $\Rightarrow$ $\langle {{LLI}_{i,EP}=LLI}_{i,1}-{C}_{i,EP}\rangle$ $\Rightarrow$ $\langle {LLI}_{i+\mathrm{1,1}}={LLI}_{i,EP}\rangle$ $\Rightarrow$ $\langle {LLI}_{i+1,j}={LLI}_{i+\mathrm{1,1}}-{C}_{i+1,j}\rangle$ (EP is the ending point). Eventually, we use Eq. (2) to obtain the overall data. Note that the value of ${LLI}_{\mathrm{1,1}}$ is still unknown here; it shall be determined by statistical features, such as the ‘zero mean’ condition, of all the measuring data.

1 $$\begin{aligned}LLI _{i,j}&={M}_{i}-{f}_{i,j}-{L}_{j}\times \sin {\theta }_{i}-{G}_{i,j} \\ &={LLI}_{i,1}-{C}_{i,j} \end{aligned}$$

where ${M}_{i}={LLI}_{i,1}+{f}_{i,1}$ and ${C}_{i,j}$ are the measurable data, which are given by ${{C}_{i,j}=f}_{i,j}+{L}_{j}\times \mathrm{sin}{\theta }_{i}+{G}_{i,j}-{f}_{i,1}$; the subscript $i,j$ represents the $j{\text{-}}th$ measuring point of the $i{\text{-}}th$ segment, and ${L}_{j}$ is the distance between the measuring point and the starting point of each segment.

2 $$\begin{array}{c}{LLI}_{i,j}={LLI}_{\mathrm{1,1}}-\sum \limits_{m=1}^{i-1}{C}_{m, EP}-{C}_{i,j}\end{array}$$

where $EP$ is the total number of measuring points in one segment.

The above measuring process clearly indicates that in the same segment, the LLI value at different measuring points is measured under the same reference (formed by the level bar). More importantly, the difference between the consecutive baselines (i.e., ${M}_{i}$ and ${M}_{i+1}$) can be eliminated mathematically at the boundary point. That is, the measuring reference of the ICM is mathematically constant, thereby ensuring that the actual waveform of LLI can be reflected and avoiding distortion from an excessive enlargement or reduction of the amplitude in the frequency domain during data recovery. Therefore, without considering the measuring error, the theoretical value of the ‘transfer function’ of ICM is constant at 1. With the consecutive data of inclination and the uneven distance, we can easily obtain the original distribution curves of the LLI by using Eqs. (1) and (2).

### Relationship with CMM

To further verify the ICM in practice, the results from CMM systems are considered. It is well known that the main weakness of CMM is the fluctuation of its transfer function. Although different parametric settings will yield various transfer functions, it is almost impossible to find a stable function using this approach. Typically, an appropriately designed filter, whose magnitude response is reciprocal to that of the transfer function, could be applied to address this effect. Recently, a novel restoration method was developed from the multipoint chord reference (MCR) system^24,25^, which provides an excellent solution for obtaining the true waveform from the signal measured by the CMM.

**Figure 4:**
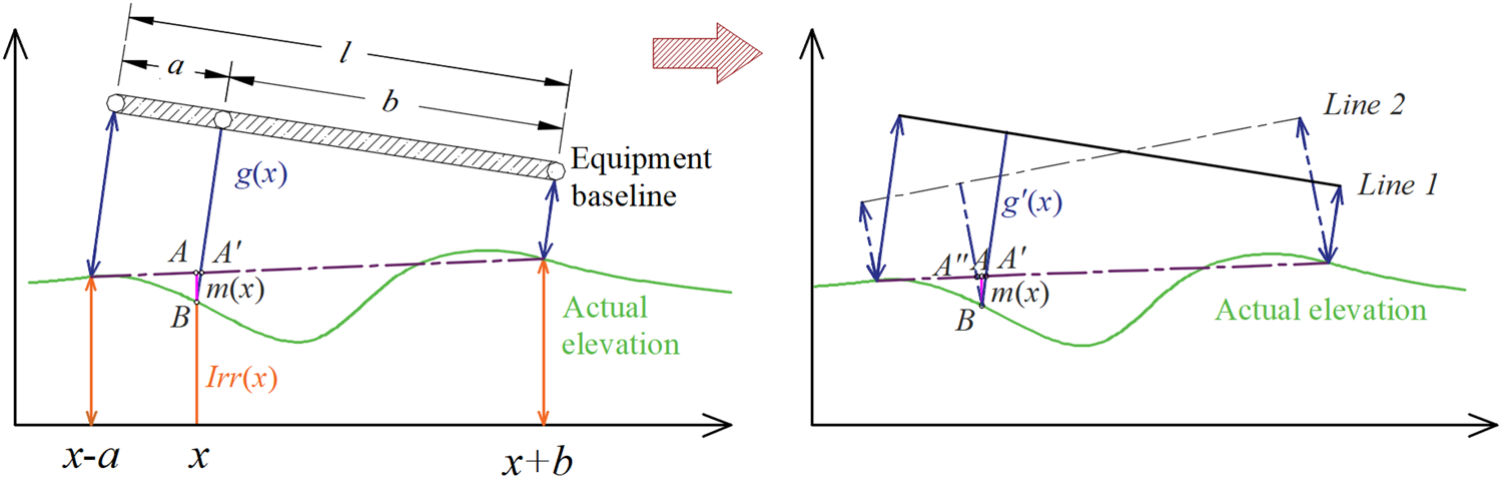
Measuring principle of the three-point offset method—one of the CMM systems.

Taking the CMM system with three testing points and unequal spacing as an example, let us first review the principle of CMM, as shown in Fig. 4. Because the magnitude of track irregularity (millimetre scale) is much lower than the chord length (metre scale), we have ${l}_{A{^{\prime}}B}\approx {l}_{AB}$, thereby obtaining Eq. (3). Similarly, for MCR systems ($a=b=l/2$), we have Eq. (4).

3 $$\begin{array}{c}m\left(x\right)=Irr\left(x\right)-\frac{b}{l}Irr\left(x-a\right)-\frac{a}{l}Irr\left(x+b\right)\end{array}$$

4 $$\begin{array}{c}m{^{\prime}}\left(x\right)=Irr\left(x\right)-\frac{1}{2}Irr\left(x-\frac{l}{2}\right)-\frac{1}{2}Irr\left(x+\frac{l}{2}\right)\end{array}$$

where $m\left(x\right), m{^{\prime}}(x)$ is the measured value of CMM and MCR, respectively, $Irr(x)$ is the actual track irregularity, and $a,b,l$ are the chord length parameters.

Based on Eq. (3) and Fig. 4, the inclination of the equipment baseline of CMM formed by the endpoints will not affect the value of $m(x)$. Furthermore, it can be found that the ICM and CMM data are highly correlated. Assume that the sample spacing in the ICM (${\Delta =L}_{j+1}-{L}_{j}$) is equal to the measurement interval of MCR ($l/2$). We then have ${LLI}_{i,j}={LLI}_{i,1}-{C}_{i,j}$, ${LLI}_{i,j-1}={LLI}_{i,1}-{C}_{i,j-1}$, and ${LLI}_{i,j+1}={LLI}_{i,1}-{C}_{i,j+1}$ when using the ICM, while $m\left(j\right)=Irr\left(j\right)-\left[Irr\left(j-1\right)+Irr\left(j+1\right)\right]/2$ is obtained when using MCR. Then, the relationship between these two methods can be described by Eq. (5). As shown in this equation, with the data measured by ICM, we can still obtain a dataset that can be adopted in MCR or other CMM systems once the sample space is adjusted to be consistent.

5 $$\begin{array}{c}m\left(j\right)=\frac{1}{2}\left({f}_{i,j-1}+{f}_{i,j+1}\right)-{f}_{i,j}+\frac{1}{2}\left({G}_{i,j-1}+{G}_{i,j+1}\right)-{G}_{i,j}\end{array}$$

### Trend extraction method

The measured track irregularity data usually contain local outlier values and global trend items because of the manual calibration, temperature drift of sensors, data transmission, etc.^16^. While using the ICM, the process of obtaining the cumulative sum of the correctional value of inclination (i.e., ${\sum }_{m=1}^{i-1}{C}_{m, End}$ in Eq. (2)) and theoretical value ${G}_{i,j}$ may result in a low-frequency and high-amplitude trend, thereby introducing errors in subsequent spectral analysis. In other words, the distribution of the measurement errors along the span is also long-term compared to its own variation. Compared to the track irregularity, these errors are unlikely to vary independently. Moreover, the composition and causes of the trend components are too complex to identify immediately. Therefore, it is necessary to study the method of trend extraction.

Conventional methods of trend extraction include high-pass filtering (HPF), the least squares method (LSM), and wavelet decomposition (WD). The main limitation of these methods lies in the priority of the algorithm. To minimize the interference of various human factors, such as selecting the basis function and cut-off frequency (or wavelength), an adaptive method based on the complete ensemble empirical mode decomposition with adaptive noise (CEEMDAN)^26^ and the Hilbert marginal spectrum (HMS)^27^ is introduced in this work. In empirical mode decomposition (EMD), which is a well-known approach, the basis function is not assumed, and the signal is decomposed into IMFs (intrinsic mode functions) based entirely on the signal characteristics. Nevertheless, the presence of mode mixing^28^ while using EMD would affect the validity of IMFs from a global perspective, thereby affecting the judgement of trend components. CEEMDAN was developed from EMD, and it eliminated mode mixing well, thereby providing excellent applicability in trend extraction. For the discrimination part, the HMS was proven effective in practice^29^ compared to conventional methods that adopt statistical parameters, including the correlation coefficient and ratio of mean IMF values.

**Figure 5:**
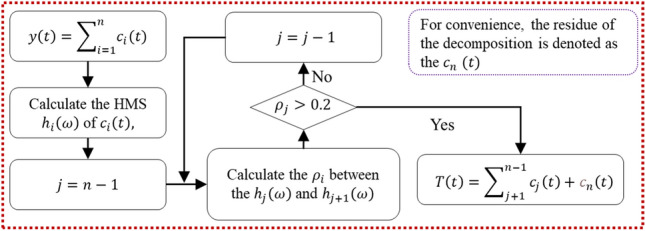
Identification process of the HMS-based method.

The general principle of this integrated method is to decompose the original signal into IMFs using CEEMDAN and then distinguish the trend components by calculating the correlation coefficient (denoted as ${\rho }_{i}$) of their HMSs, thereby evaluating the separation degree of the adjacent IMFs, as illustrated in Fig. 5. The calculation of HMS has been well described in the literature^27^. In addition, the ${\rho }_{i}$ values of the HMSs are calculated in a discrete form to meet the actual measuring conditions; see Eq. (6). A specific example will be given in the next chapter.

6 $$\begin{array}{c}{\rho }_{i}=\left|\frac{\sum_{m}\left({h}_{i}\left(m\Delta \omega \right)-{\overline{h} }_{i}\right)\left({h}_{i+1}\left(m\Delta \omega \right)-{\overline{h} }_{i+1}\right)}{\sqrt{\sum_{m}{\left({h}_{i}\left(m\Delta \omega \right)-{\overline{h} }_{i}\right)}^{2}\sum_{m}{\left({h}_{i+1}\left(m\Delta \omega \right)-{\overline{h} }_{i+1}\right)}^{2}}}\right|\end{array}$$

where ${h}_{i}\left(m\Delta \omega \right)$ is a discrete form of ${h}_{i}\left(\omega \right)$ that adapts to the measured signal.

### Estimation and fitting of the PSD for track irregularity

The measured track irregularity, which is affected by many factors, is not a strictly stationary and random signal in terms of engineering practice. Generally, the PSD function is applied to describe these characteristics. In railway systems, the classic PSD estimation methods, such as the ‘periodogram’ and the ‘Welch method’ (see Eqs. (7) and (8)), have been widely used. Classic methods are generally based on the Fourier transform (FT), which means they cannot satisfy the frequency resolution and variance performance at the same time. The method of parametric estimation, such as the auto-regressive (AR) model, however, generally gives better frequency accuracy and lower variance because it eliminates the effect of FT and makes more reasonable assumptions about the data outside the window of the spectral calculation. To explain the establishment of an AR model, let us consider a linear system with a transfer function $H\left(z\right)$, in which the input sequence $\left\{u\left(n\right)\right\}$ and the output sequence $\left\{x\left(n\right)\right\}$ are related by the difference equation Eq. (9). When the value of $\left\{{b}_{i}\right\}$, except ${b}_{0}=1$, is zero, the process is a pure autoregressive (AR) model, and we can estimate its PSD by using Eq. (10). Therefore, to estimate the PSD, one only needs to estimate the parameters $\left\{{a}_{i}\right\}$ and the corresponding variance ${\sigma }^{2}$.

7 $$\begin{array}{c}\widehat{P}\left({f}_{m}\right)=\frac{1}{N\Delta t}{\left|\begin{array}{c}\sum_{n=0}^{N-1}x\left[n\right]\cdot {e}^{\frac{-j2\pi mn}{N}}\end{array}\right|}^{2} m=\mathrm{0,1},2,\ldots ,N-1\end{array}$$

where $\widehat{P}\left({f}_{m}\right)$, ${f}_{m}$, $N$, $\Delta t$, and $x\left[n\right]$ are the estimated value, sampling frequency, number of samples, sampling period, and the input of a discrete-time series, respectively.

8 $$\begin{array}{c}{\widehat{P}}_{Welch}\left(\omega \right)=\frac{1}{LMU}\sum_{i=1}^{L}{\left|\begin{array}{c}\sum_{n=0}^{M-1}{x}_{N}^{i}\left(n\right)\cdot d\left(n\right)\cdot {e}^{-j\omega n}\end{array}\right|}^{2}\end{array}$$

where ${x}_{N}\left(n\right)$, ${\widehat{P}}_{Welch}\left(\omega \right)$, $\omega$,$L$, $M$,$U$, and $d\left(n\right)$ are the input data, estimation of ${x}_{N}\left(n\right)$, angular frequency, number of segments, length of each segment, normalization factor given by $U=\frac{1}{M}\sum_{n=0}^{M-1}{d}^{2}\left(n\right)$, and the window (function), respectively.

9 $$\begin{array}{l}\left\{\begin{array}{l}x\left(n\right)=-\sum_{k=1}^{p}{a}_{k}x\left(n-k\right)+\sum_{k=0}^{q}{b}_{k}u\left(n-k\right)\\ H\left(z\right)=\sum_{m=0}^{q}{b}_{m}{z}^{-m}/\sum_{m=0}^{p}{a}_{m}{z}^{-m}\end{array}\right. \end{array}$$

10 $$\begin{array}{c}{PSD}_{AR}\left({e}^{j\omega }\right)={\sigma }^{2}/{\left|1+\sum_{k=1}^{p}{a}_{k}{e}^{-j\omega k}\right|}^{2}\end{array}$$

where $z$ is the Z-transformation and $\sigma$ is the standard deviation.

In this work, three effective algorithms to estimate the parameters of the AR model are considered: (i) The autocorrelation method, which uses the ‘Levinson–Durbin recursion’ to solve the ‘Yule–Walker equation’ that connects $\left\{{a}_{i}\right\}$ and the autocorrelation coefficients of the signal. (ii) The Burg algorithm, which minimizes the sum of the forwards and backwards prediction error energies of the output sequence $\left\{x\left(n\right)\right\}$ while satisfying the LD recursion. (iii) The modified covariance algorithm, which adopts a similar process as the Burg algorithm to improve the frequency resolution and eliminate the constraint of the Levinson–Durbin recursion and solves the canonical equation by approaches such as Cholesky factorization or Marple’s algorithm. The specific calculation process of the above algorithms can be found in the literature^30,31^.

The estimated PSD of track irregularity generally conserves its characteristics and information from the frequency domain. However, the estimated PSD curve, in its current state, is not applicable for the potential research conducted by other researchers until it is expressed by a specific equation. For instance, the equation ${S}_{1}\left(f\right)=\alpha /\left({f}^{n}+{\beta }^{n}\right)$ was proposed based on the Japanese monorail and applied in many studies. However, ${S}_{1}\left(f\right)$ is a conventional formula oriented from road systems, and it overestimates the impact of the longwave track irregularity of the original data of track irregularity. Moreover, there is deficiency in its original data, as we have already discussed in the Introduction section. Here, we propose an improved model ${S}_{2}\left(f\right)$ to fit the estimated PSD of the LLI data of MTTS. Additionally, the applicability of the piecewise power function ${S}_{3}\left(f\right)$ and polynomial model ${S}_{4}\left(f\right)$ (in logarithm) adopted by railway systems is examined, as shown in Eq. (11).

11 $$\begin{array}{l}\left\{\begin{array}{ll}{S}_{1}\left(f\right)=\frac{\alpha }{{f}^{n}+{\beta }^{n}}& \quad {S}_{2}\left(f\right)=\frac{\alpha f}{{f}^{n}+{\beta }^{n}}\\ {S}_{3}\left(f\right)=\frac{\alpha }{{f}^{n}}& \quad \ln{S}_{4}\left(f\right)=\sum_{i=0}^{n}{\alpha }_{i}\mathrm{ln}{f}^{i}\end{array}\right. \end{array}$$

where $f$ is the spatial frequency in 1/m; $\alpha$,$\beta$, and $n$ are values to be fitted.

## Data preparation

### Simulation and verification

**Figure 6:**
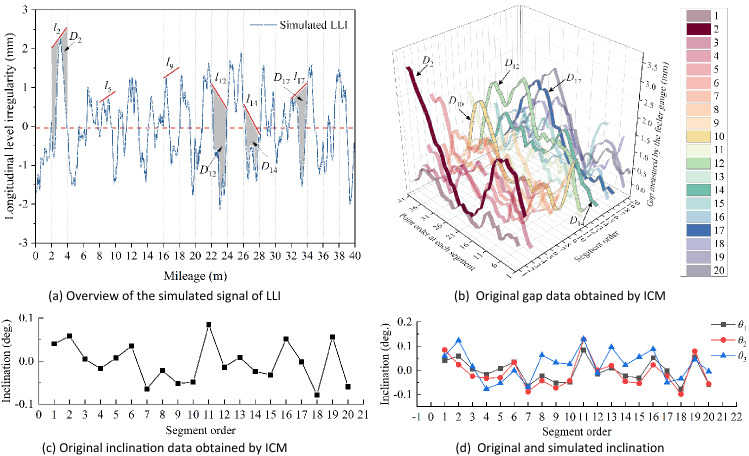
Identification process of the HMS-based method.

To further verify the ICM, the results obtained by MCR are considered using simulations. According to Eq. (5), the data measured through ICM could be analysed as the measured value of MCR. To simplify the calculation process, the designed slope of the simulated track line is set to zero, thereby eliminating the influence of ${G}_{i,j}$. Figure 6a shows the distribution of the simulated LLI curve with a total length of 40 m, and each segment has a length of 2 m. Based on the ICM process, the measurement baseline of the ‘level bar’ adopted in ICM is simulated by constructing a straight line that is higher than all other points in the same segment; see ${l}_{i}$ in Fig. 6a. Next, the distance between ${l}_{i}$ and the simulated LLI curve (${D}_{i}$) is measured and taken as the value measured by the feeler gauge (${f}_{i,j}$ in Eq. (5)) in ICM, as shown in Fig. 6b. In addition, the inclination of ${l}_{i}$ is calculated and taken as the value measured by the inclinometer in ICM, as shown in Fig. 6c. Moreover, to examine the effectiveness of the proposed trend extraction on eliminating the cumulated error, let the original data be ${\theta }_{1}$ and define two sequences given by ${\theta }_{2}={\theta }_{1}+0.05^\circ \times \mathrm{Rand}[-\mathrm{1,1}]$ and ${\theta }_{3}={\theta }_{1}+0.10^\circ \times \mathrm{Rand}[-\mathrm{1,1}]$, where $\mathrm{Rand}[-\mathrm{1,1}]$ is a random sequence between − 1 and 1, and 0.05 $^\circ$ is the nominal precision of the inclinometer we used in this work, see Fig. 6d.

To date, the data measured by the ICM have been prepared and analysed using MCR systems. Then, the distribution curves of the LLI measured by MCR can be obtained in two ways: directly measuring the versine value from the original curve (as shown in Fig. 6a) or applying Eq. (5) to the gap data measured through ICM (as shown in Fig. 6b). Apparently, these two approaches achieve the same results. Next, the distribution curve of the LLI can be obtained. For ICM, we can simply apply Eq. (2) to the simulated gap and inclination data. For the MCR system, we can generally solve it using two processes^24^, including multiplying the inverse of the measurement matrix by the vectors of the measured versine ($f={T}^{-1}h$) and performing iterative computations. In addition, the offset value at the start and end point is assumed to be zero (${f}_{0}={f}_{n+1}=0$) to provide the necessary conditions for the matrix operation.

### Field tests based on MTTS

**Figure 7:**
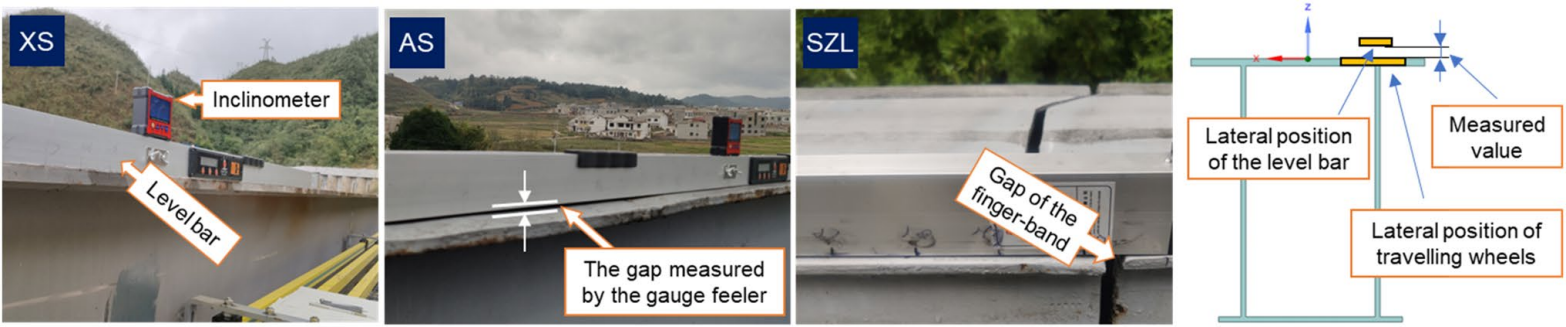
Configuration of the field tests to measure the LLI.

**Figure 8:**
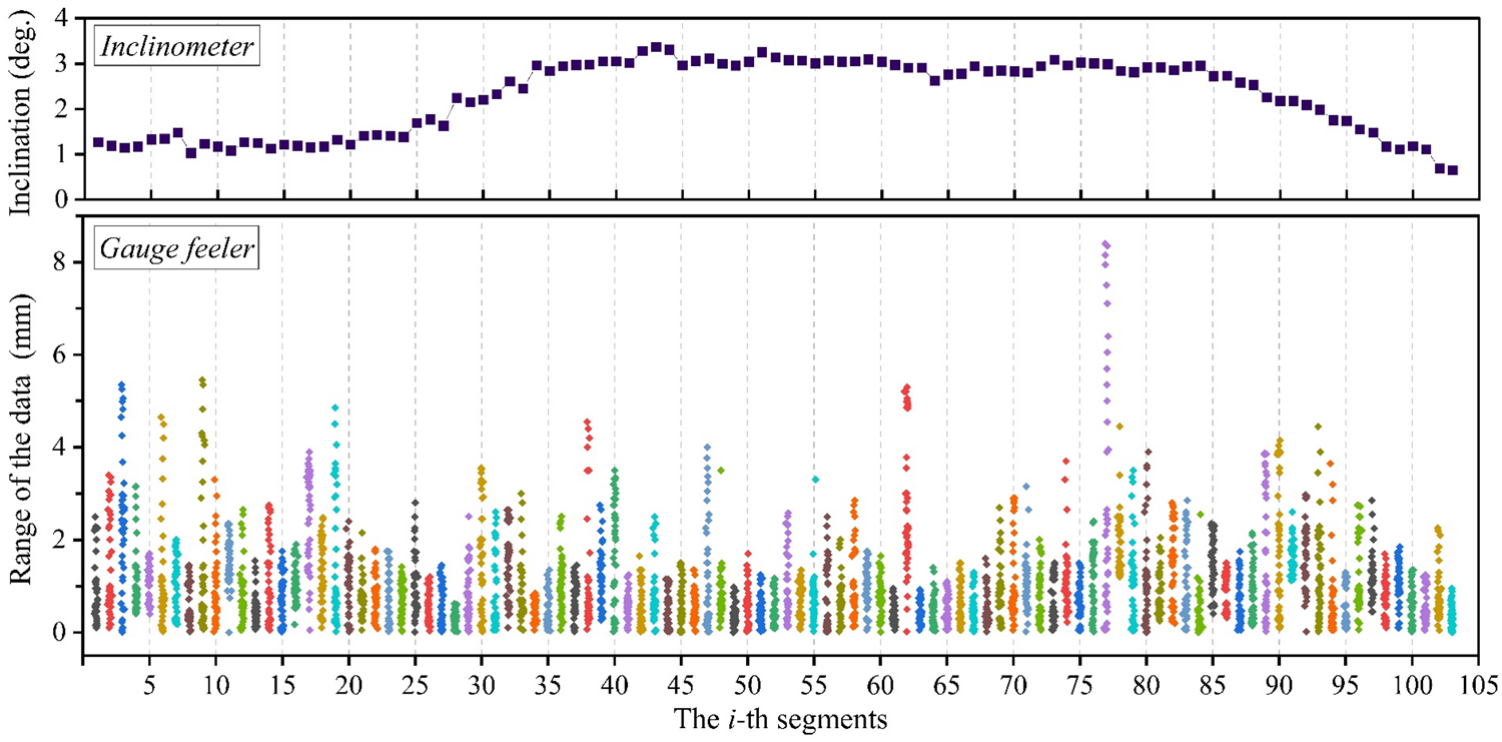
Original data measured by ICM in the XS project.

In this work, we measured the LLI of MTTS projects located in Xi–Shui (XS) and An–Shun (AS) in Guizhou Province and San–Zhao–Lun (SZL) in Jiangxi Province in China. A section form of a welded steel box is adopted for all track beams, although their geometric dimensions vary with each design case. From engineering experience, the effects induced by the lateral irregularity on the top plate are generally ignored. Hence, this work does not consider the influence of the actual lateral position on the track beam. Instead, the value measured by the feeler gauge at each point is the average in the transverse direction of the gap between the level bar and the track beam; see Fig. 7 below. Take the XS project as an example. We measured a total of 4096 points with a sample interval of 5 cm, and the original data tested by the ICM are shown in Fig. 8.

## Results analysis

### Simulation of ICM and MCR

**Figure 9:**
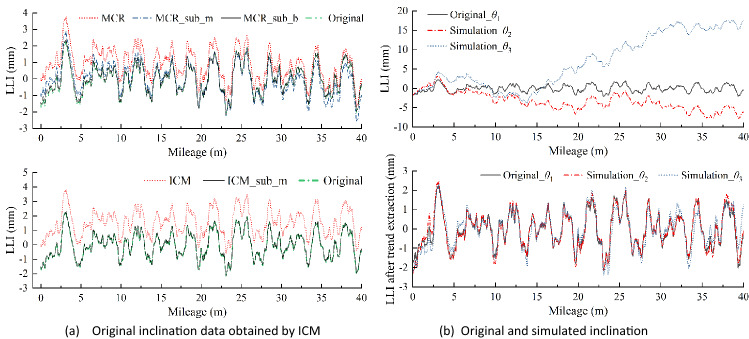
Results obtained by different methods. Note that the subscripts ‘$sub\_m$’ and ‘$sub\_b$’ represent subtracting the mean value and baseline of the original curve, respectively.

Simulation cases were introduced in the former section. Here, the simulation results will be discussed in detail. To verify the effectiveness of ICM, the results from the recognized MCR system are compared. Figure 9a illustrates the results of the distribution curve of LLI obtained by MCR and ICM. As shown, overall shifting is observed in the distribution curve obtained by MCR, which is typical and highly correlated to the predefined conditions of the LLI sequence, especially for such a short sequence. Nevertheless, this issue can be addressed by eliminating the inherent baseline of the measured curve—another trend extraction method. The distribution curve obtained by ICM is also influenced by the assumed value of the start point. Nevertheless, this effect can be simply addressed by subtracting the mean value of the curve. Obviously, the results obtained by ICM and MCR are highly consistent with each other and the original data. The root-mean square (RMS) values of the residual sequence between the measured and original data, defined as $\left\{{\Delta }_{1}\right\}={\mathrm{Curve}}_{MCR\_sub\_b}-{\mathrm{Curve}}_{Ori}$ and $\left\{{\Delta }_{2}\right\}={\mathrm{Curve}}_{ICM\_sub\_m}-{\mathrm{Curve}}_{Ori}$), are 0.06 mm and 0.03 mm, respectively.

Furthermore, the distribution curves obtained through ICM under the different level errors of inclination data are presented in Fig. 9b. As shown, the distribution curve of the LLI is distorted due to the predefined error. Nevertheless, the LLI information, especially the relative value of the measuring points, is well preserved. This characteristic again illustrates that the inclination errors can induce strong effects, but most results in the overall shifting of the original data. In other words, the effect of extra inclination is represented on the original curve as the additional trend components. Similarly, the RMS values of the residual sequence of $\left\{{\Delta }_{3}\right\}={\mathrm{Curve}}_{\mathrm{sim}\_\theta 2}-{\mathrm{Curve}}_{Ori}$ and $\left\{{\Delta }_{4}\right\}={\mathrm{Curve}}_{\mathrm{sim}\_\theta 3}-{\mathrm{Curve}}_{Ori}$ are 0.16 mm and 0.36 mm, respectively. On this basis, the supplement of the proposed trend extraction method is proven effective. In summary, the above MCR results strongly validate the effectiveness of ICM; in addition, the proposed trend extraction method significantly improves the robustness of ICM.

### Results of the distribution curve in the field tests

**Figure 10:**
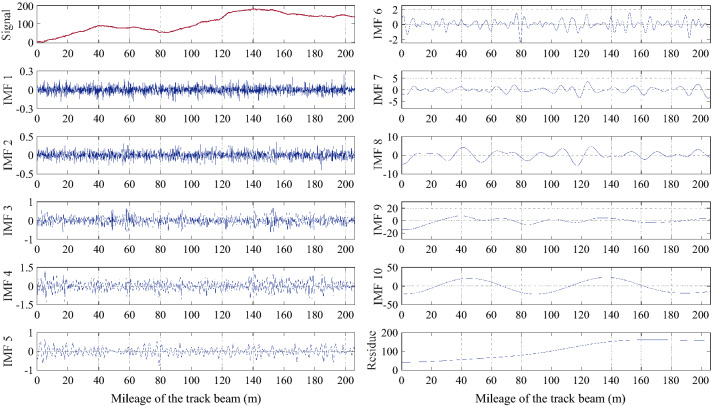
Results decomposed using CEEMDAN.

**Figure 11:**
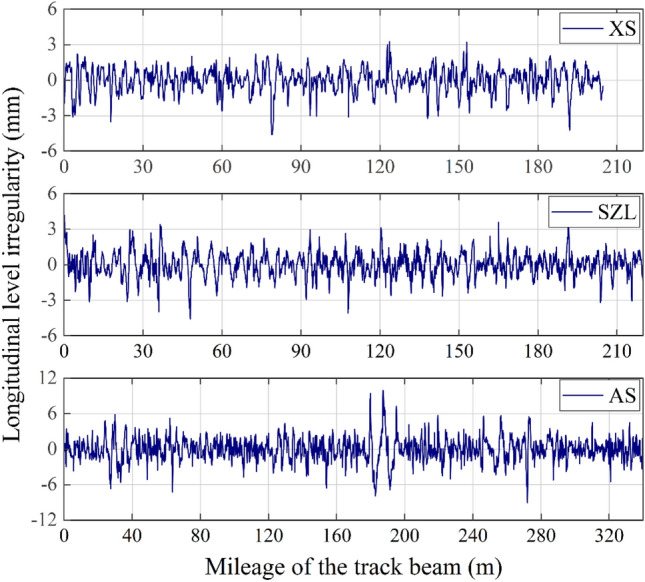
Distribution curves of the LLI for MTTS projects.

Previous simulation results have profoundly verified the proposed methods. In this subsection, the results of LLI measured in different MTTS projects by using ICM are discussed. As we have described in the preceding, the distribution curve of LLI can be obtained using Eq. (2). The field-tested inclination data inevitably contain measurement errors, thereby distorting the distribution curve. This also means that trend extraction is needed. As an example, Fig. 10 presents the LLI curve for the XS project and the corresponding decomposition results obtained using CEEMDAN. Clearly, the original signal (i.e., the distribution curve) is decomposed into ten IMFs and one residue. After calculating the HMS of the IMFs, the sequence of correlation coefficients $\left\{{\rho }_{i}\right\}$ of adjacent HMSs is then obtained (see Table 1). From Table 1, it is easy to determine that the $\left\{{\rho }_{i}\right\}$ of the XS project becomes much larger than the front and the empirical value of 0.2 after ${\rho }_{7}$. Therefore, the trend components should be the sum of IMF 7 to the residue in this case. The above results show that CEEMDAN is appropriate for the decomposition of LLI signals, and the HMS-based method can intuitively reflect the correlation of the HMS of adjacent components, thereby providing significant discrimination between the trend components and general components. After eliminating the trend from the original signal, we can finally obtain the distribution curve of the LLI for the XS project; by the same token, the distribution curves of the other projects can be obtained (see Fig. 11).

**Table 1:** Correlation coefficients of the HMS of the adjacent IMFs.

Order	${\rho }_{1}$	${\rho }_{2}$	${\rho }_{3}$	${\rho }_{4}$	${\rho }_{5}$	${\rho }_{6}$	${\rho }_{7}$	${\rho }_{8}$	${\rho }_{9}$	${\rho }_{10}$
Results	0.5798	0.3910	0.2007	0.1143	0.0680	0.0868	0.3947	0.6693	0.7158	0.9358

### Results of spectral analysis in field tests

**Figure 12:**
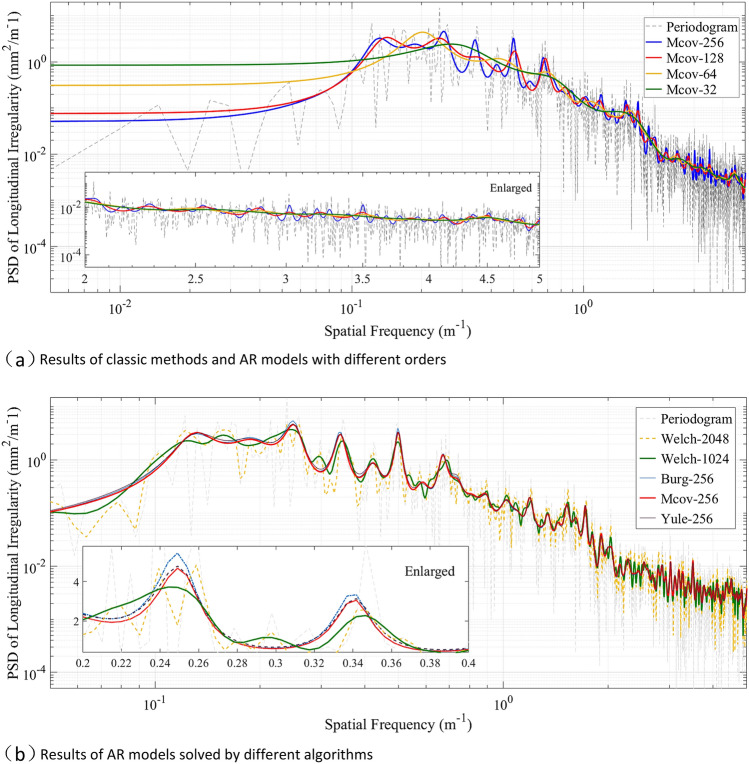
Comparison of estimation methods: (**a**) and (**b**). Note the ‘Mcov-256’ indicates the modified covariance algorithm with AR order of 256, ‘Burg’ is the Burg algorithm, and ‘Yule’ is the autocorrelation method.

Similarly, taking the LLI of the XS project as an example, spectral estimation and its fitting are conducted on the distribution curve of LLI. First, the previously mentioned methods are applied to the data for the XS project, and the results are shown in Fig. 12. From Fig. 12a, it can be observed that the AR model shows better performance on variance compared to the ‘periodogram.’ As the parametric order increases from 32 to 256, spectral peaks appear in succession, and the sharpness of the spectrum improves. As shown in Fig. 12b, compared with the ‘AR model,’ smoother peak are obtained using the ‘Welch method’ with a window length of 1024, which is a quarter of the length of the input data. If we increase the window length to 2048 (i.e., half of the input data), the performance of the estimate variance becomes much poorer. Moreover, Fig. 12b indicates that the PSD estimated by the AR model solved by different algorithms has great consistency in this case. Among these algorithms, the modified covariance method has the best performance in terms of spectral sharpness, while the amplitude peak of the Burg algorithm is slightly higher than that of the other algorithms.

**Figure 13:**
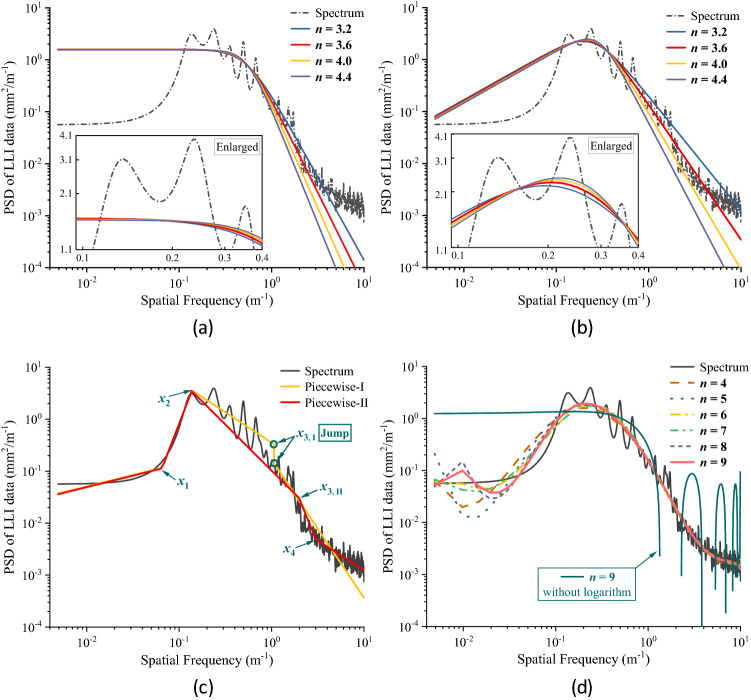
Fitting results of PSD curves: (**a**) to (**d**) are fitted by ${S}_{1} (f)$ to ${S}_{4} (f)$, respectively.

In summary, the AR model is more suitable for the spectral estimation of LLI data than the classic methods. Meanwhile, it should be noted that the selection of order primarily affects the performance of the AR model. Ordinarily, a higher order tends to provide better resolution in the frequency domain. Since the 1970s, the performance of different criteria, including the Akaike information criterion (AIC), final prediction error (FPE), and their modified criteria^32^, in determining the model order has been studied. However, the applicability of these criteria in the estimation of track irregularity is not clear thus far. In fact, the orders calculated by these criteria are too small (generally less than 20) to obtain a satisfactory resolution of the PSD of LLI in practice. Based on this, we suggest adopting looser criteria and increasing the recommended order given by traditional criteria to obtain a better frequency resolution. Figure 13 depicts the fitting results for the XS project. The coefficient of determination (${R}^{2}$, ratio of explained sum of squares (ESS) to total sum of squares (TSS)) is used to evaluate the performance of the fitting process (see Table 2).

**Table 2:** Results of ${R}^{2}$ of the fitting.

Model	Coefficient of determination (${R}^{2}$)							
${S}_{1}\left(f\right)$	*n* = 3.2	*n* = 3.4	*n* = 3.6	*n* = 3.8	*n* = 4.0	*n* = 4.2	*n* = 4.4	*n* = 4.6
	0.639	0.642	0.643	0.644	0.644	0.644	0.644	0.644
${S}_{2}\left(f\right)$	*n* = 3.2	*n* = 3.4	*n* = 3.6	*n* = 3.8	*n* = 4.0	*n* = 4.2	*n* = 4.4	*n* = 4.6
	0.815	0.819	0.820	0.820	0.818	0.816	0.812	0.809
${S}_{3}\left(f\right)$	Fitting-1: 0.836				Fitting-2: 0.502			
${S}_{4}\left(f\right)$	*n* = 2	*n* = 3	*n* = 4	*n* = 5	*n* = 6	*n* = 7	*n* = 8	*n* = 9
	0.443	0.575	0.577	0.551	0.548	0.585	0.443	0.575

As the result shows, the modified fractional model ${S}_{2}\left(f\right)$ fits the spectrum better than ${S}_{1}\left(f\right)$: the shape and peak value of the fitting curves are more consistent with the original PSD curve; in addition, the ${R}^{2}$ values of the curves fitted by ${S}_{2}\left(f\right)$ are apparently higher than those fitted by ${S}_{1}\left(f\right)$ (see Fig. 13a,b). In addition, the curves fitted by ${S}_{3}\left(f\right)$ have the highest value of ${R}^{2}$, but the fitted curves are discontinuous (or abrupt) at the segment points, e.g., point ${x}_{3, i}$ of ‘Piecewise-I’ (see Fig. 13c). However, the solving process or the selection of segment points either becomes much more difficult or empirical. The value of ${R}^{2}$ for ${S}_{4}\left(f\right)$ does not always increase as the equation order increases, but the overall values are lower than those fitted by ${S}_{\mathrm{1,2},3}\left(f\right)$. Note that the ${R}^{2}$ here is the determination coefficient between the original data and ${S}_{4}\left(f\right)$, not $\mathrm{ln}{S}_{4}\left(f\right)$. The reason for using a previously logarithmic transform is that the routine polynomial inevitability produces interval oscillation values near zero, which is inconvenient for spectral analysis (see Fig. 13d). Frankly, it seems that ${S}_{4}\left(f\right)$ performs better in the low-frequency region than ${S}_{2}\left(f\right)$. Nevertheless, the measured track irregularity signal may underestimate the amplitude in that area because of the process of trend extraction and the assumption of Gaussian stationarity. Therefore, without the support of more advantages, we believe that it is feasible to adopt ${S}_{2}\left(f\right)$.

In general, the fractional model has great adaptability and a high coefficient of determination in the fitting of the estimated PSD. Compared with the Japanese model ${S}_{1}\left(f\right)$, the proposed model ${S}_{2}\left(f\right)$ is more accurate—according to Table 2, the ${R}^{2}$ of ${S}_{2}\left(f\right)$ is more than 25% higher than that of ${S}_{1}\left(f\right)$. Considering the deficiency of the polynomial and piecewise power functions and the wide application of fractional models in railway, road, and monorail systems, ${S}_{2}\left(f\right)$ is suggested for the fitting of the estimated PSD of the LLI data from monorail systems.

### Evaluation of the PSD of measured LLI

**Figure 14:**
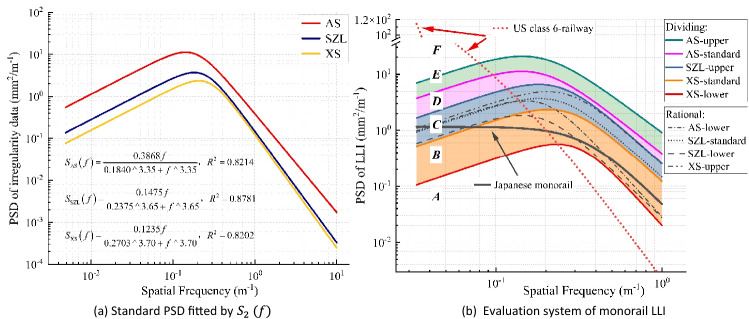
PSDs of the LLI in different MTTS projects.

Once we have sufficient measured data on track irregularity, a standardized spectrum can be obtained in terms of types of track irregularity, line levels, and so on. The lower the position of the PSD curve on the ordinate axis is, the better the conditions of the track irregularity and vice versa. Figure 14a illustrates the fitting results of the PSD curves of MTTS projects by using ${S}_{2}\left(f\right)$. Clearly, the status of the track beam in the XS project is the best among all projects, followed by the SZL and AS projects, which are consistent with their construction and operation statuses. In fact, the XS project was not put into operation during the field measurement, while the SZL project was in normal operation for over two years and the AS project was in a poor state of repair—there were visible defects on the surface of the track beam, such as corrosion and pits, due to disrepair.

Furthermore, if we fully consider the upper and lower boundaries of the estimated PSD curves, we can obtain fitted PSD curves with a broader range, thus making it more reasonable to establish the classification system based on the relative height of the spectral lines. Specifically, we first obtain the lower and upper envelopes of the estimated PSD of the XS, SZL, and AS engineering projects and then fit the six envelopes by using ${S}_{2} (f)$. With the standard PSD of three engineering projects, we plot nine PSD curves with different overall spectral heights. Table 3 presents the fitting results of the lower and upper envelopes of the estimated PSD based on ${S}_{2}\left(f\right)$.

**Table 3:** Parametric values fitted by ${S}_{2}\left(f\right)$.

Type	PSD curve	Fitting parameter		
		$\alpha$	$\beta$	$n$
Diving spectrum	AS-upper	0.9198	0.1841	3.21
	AS-standard	0.3868	0.1840	3.35
	SZL-upper	0.2565	0.2358	3.65
	XS-standard	0.1235	0.2703	3.70
	XS-lower	0.0202	0.3068	4.28
Rational spectrum	AS-lower	0.0287	0.1972	4.27
	SZL-standard	0.1475	0.2375	3.65
	SZL-lower	0.0278	0.1863	3.84
	XS-upper	0.2486	0.2660	3.69

Of course, this kind of qualitative method has certain limitations: the PSD of the actual track irregularity covers a wide range of frequencies (or wavelengths), so the relative height of the spectral line may not be the same in different regions. The supplement of a quantitative method^33^ has been proven to be a reasonable way to solve this problem. For instance, we can judge the state of the LLI based on its mean square value, which is the integral of the PSD curve (i.e., the area under the spectral line of LLI data) in terms of mathematics. The numerical interval of the fitted PSD curves is given in Table 4, and the status of the LLI of MTTS is classified into six areas by five diving spectral lines, i.e., the ‘AS upper,’ ‘AS standard,’ ‘SZL-upper,’ ‘XS-standard,’ and ‘XS lower’ lines. Thus far, the evaluation system has been established. With any measured or simulated LLI data of monorail systems, the status of the track beam can be evaluated expediently. Taking the PSD function of the Japanese monorail and the US Federal Railroad Administration (FRA) with class 6 as examples, we draw their PSD curves and the curves fitted in this work in Fig. 14b.

**Table 4:** Numerical values of the evaluation system.

Wavelength range (m)	Numerical interval of all grades (mm^2^)					
	F	E	D	C	B	A
1 to 30	> 6.8857	(3.5058, 6.8857]	(2.3163, 3.5058]	(0.9270, 2.3163]	(0.2171, 0.9270]	< 0.2171
0.5 to 60	> 14.2291	(7.1588, 14.2291]	(4.6918, 7.1588]	(1.8896, 4.6918]	(0.4327, 1.8896]	< 0.4327

Clearly, the spectral line of the Japanese spectrum mainly falls between level B and level C of the evaluation system of MTTS. The mean square value of the Japanese spectrum is 0.44 in the wavelength range of 1–30 m and 0.93 in the wavelength range of 0.5–60 m, indicating an overall status of level B. For the spectrum of the US railway, the shape of its PSD curve is quite different from that of monorail systems, and its spectral value decreases sharply with increasing spatial frequency. Specifically, the value is higher than ‘AS-upper’ in the range of wavelengths greater than 12.5 m and lower than ‘XS-lower’ in the range of wavelengths less than 3.87 m. The mean square values of the US spectrum in these two ranges are 2.78 and 13.11, respectively, which fall between level D and level E. Now, we can conclude that the track beam of the Japanese monorail is in good status, which also means that while adopting the Japanese spectrum in the research of vehicle systems, the impact of track irregularity is likely to be underestimated. Due to the significant difference in the characteristics of the PSD curve, the spectrum of the US railway is not appropriate for the study of monorail systems.

## Discussion

In this work, we propose the ICM and measure the LLI of MTTS projects. The presented results from simulation and field tests strongly verify the applicability and robustness of the proposed methods. Nevertheless, further studies should be conducted on the performance of ICM. For instance, more precise or automated instruments can be applied while adopting the ICM, although the equipment we used can satisfy the requirement in this work; the error performance under various measuring conditions shall be investigated.

Furthermore, the presented PSDs of LLI are undoubtedly much more abundant than the existing and only Japanese data. However, the measured samples are still deficient for establishing a comprehensive evaluation system of the LLI; moreover, studying the influence of LLI on the dynamic response of the monorail track beam system would contribute to this issue. Considering the manuscript length, the above work has not been included in this work.

## Conclusion

Based on MTTS projects, detailed measurements and spectral analyses of the LLI of the track beam are carried out in this work for the first time. First, the proposed methods are verified by comparing the simulation results obtained by ICM with MCR. Then, the LLI of three engineering projects is measured through field tests. The calculation results of the PSD of LLI can be applied and are essential for subsequent research on the dynamic response of monorail systems. The main conclusions are as follows.

- By measuring the inclination of each measurement segment, the proposed ICM can be used to construct a corrected reference during the measurement of LLI. With continuous measuring processes, a constant reference for the overall program is established, and hence, the advantages of the waveform validity and broader range of wavelengths of the tested signal are achieved. The theoretical analysis and simulation results from chord-based methods strongly verify the effectiveness and demonstrate the robustness of ICM.
- It is observed that the cumulative errors of ICM, especially the measuring error of inclination, generally induce trend components to the LLI curve. The calculation results indicate that the combined use of CEEMDAN and HMS is conducive to adaptive signal decomposition and significant distinction of trend components, thereby providing remarkable robustness to ICM.
- The estimation results clearly show the applicability and advantages of the AR model to LLI data when adopting a loose criterion. The comparison of various fitting models indicates that the proposed model ${S}_{2}\left(f\right)=\alpha f/\left({f}^{n}+{\beta }^{n}\right)$ provides a higher coefficient of determination and better shape for the fitted PSD curves, while some conventional functions may have problems in fitting convergence and data continuity. Furthermore, we employ ${S}_{2}\left(f\right)$ and fit the upper and lower envelopes of the estimated spectra and calculated the numerical intervals of PSD curves based on the mean square value, thereby establishing an evaluation system that is applicable for potential research on monorail systems.
- The comparison shows that the characteristics of railway spectra are quite different from those of the monorail. The Japanese spectrum is mainly limited by its sample size and imperfect spectral analysis. On these bases, the fitted results and evaluation framework of the monorail track spectra can be a valuable reference. Nevertheless, further studies should be conducted on the measurement effectiveness and error performance of ICM. Moreover, the influence of track irregularity, especially the LLI, in generalized monorail systems should be investigated in depth, thereby making the evaluation system more reasonable and applicable.

## Supplementary Information

Supplementary Information.

## Data Availability

The datasets generated and analysed during the current study are not publicly available due to the agreement between us and our cooperation enterprise (The Zhuzhou CRRC Special Equipment Tech. Co., LTD, China) but are available from the corresponding author on reasonable request. In addition, part of the data generated or analysed during this study are included in this manuscript (e.g., Fig. 8) and are available in Supplementary Information.
